# USP11 Promotes Endothelial Apoptosis-Resistance in Pulmonary Arterial Hypertension by Deubiquitinating HINT3

**DOI:** 10.70322/jrbtm.2025.10002

**Published:** 2025-03-24

**Authors:** Bum-Yong Kang, Jiwoong Choi, Victor Tseng, Yutong Zhao, Jing Zhao, Robert S. Stearman, Wilbur A. Lam, Viranuj Sueblinvong, Benjamin T. Kopp, Michael J. Passineau, Changwon Park, John Lister, Raymond J. Benza, Andrew J. Jang

**Affiliations:** 1 Department of Pediatrics, Division of Pulmonology, Asthma, Cystic Fibrosis, and Sleep, Emory University School of Medicine, Atlanta, GA 30322, USA; 2 Department of Medicine, Division of Pulmonary, Allergy, Critical Care, and Sleep Medicine, Emory University School of Medicine, Atlanta, GA 30322, USA; 3 Atlanta Veterans Healthcare System, Decatur 30033, GA, USA; 4 Division of Pulmonary, Critical Care, and Sleep Medicine, Department of Internal Medicine, University of Kansas School of Medicine, Kansas City, KS 66160, USA; 5 Bioengineering Program, University of Kansas, Lawrence, KS 66045, USA; 6 Respiratory Medicine, Ansible Health, Mountain View, CA 94043, USA; 7 Department of Physiology and Cell Biology, The Ohio State University, Columbus, OH 43210, USA; 8 Department of Medicine, University of Indiana, Indianapolis, IN 46202, USA; 9 Wallace H. Coulter Department of Biomedical Engineering, College of Engineering, Georgia Institute of Technology, Atlanta, GA 30332, USA; 10 Cardiovascular Institute, Allegheny Health Network, Pittsburgh, PA 15212, USA; 11 Department of Molecular and Cellular Physiology, Louisiana State University Health Science Center, Shreveport, LA 71103, USA; 12 Department of Medicine, Division of Hematology and Cellular Therapy, Allegheny Health Network Cancer Institute, Pittsburgh, PA 15224, USA; 13 Department of Medicine, Drexel University College of Medicine, Philadelphia, PA 19104, USA; 14 Ichan School of Medicine, Mount Sinai Fuster Heart Hospital, New York, NY 10029, USA

**Keywords:** USP11, Deubiquitination, HINT3, BCL2, Pulmonary hypertension, Apoptosis-resistance

## Abstract

Pulmonary arterial hypertension (PAH) is a progressive, lethal, and incurable disease of the pulmonary vasculature. A previous genome-wide association study (GWAS) with Affymetrix microarray analysis data exhibited elevated histidine triad nucleotide-binding protein 3 (HINT3) in the lung samples of PAH compared to control subjects (failed donors, FD) and the positive correlations of HINT3 with deubiquitinase USP11 and B-cell lymphoma 2 (BCL2). In this study, we aim to investigate the roles and interplay of USP11 and HINT3 in the apoptosis resistance of PAH. The levels of USP11 and HINT3 were increased in the lungs of idiopathic PAH (IPAH) patients and Hypoxia/Sugen-treated mice. USP11 and HINT3 interacted physically, as shown by co-immunoprecipitation (co-IP) assay in human pulmonary arterial endothelial cells (HPAECs). HINT3 was degraded by polyubiquitination, which was reversed by USP11. Furthermore, HINT3 interacted with the anti-apoptotic mediator, BCL2. Overexpression of USP11 increased BCL2 content, congruent to elevated lung tissue levels seen in IPAH patients and Hypoxia/Sugen-treated mice. Conversely, the knockdown of HINT3 function led to a depletion of BCL2. Thus, we conclude that USP11 stabilizes HINT3 activation, which contributes to endothelial apoptosis-resistance of pulmonary arterial endothelial cells in PAH. This can potentially be a novel therapeutic target for ubiquitination modulators for PAH.

## 1. Introduction

Pulmonary hypertension (PH) is characterized by a progressive increase in pulmonary vascular resistance due to endothelial dysfunction, abnormal proliferation of pulmonary vascular endothelial cells, and vascular remodeling. Pulmonary arterial hypertension (PAH) is a progressive and incurable disease marked by pulmonary vascular occlusive remodeling leading to eventual right ventricular failure and death [1–3]. The effective 5-year survival is only ~60% amongst those with PAH [4–6].

Ubiquitin-specific proteases (USPs) are deubiquitinating enzymes involved in cell proliferation, migration, and apoptosis. Emerging evidence suggests that USPs are linked to PH pathogenesis by removing ubiquitin groups from unanchored polyubiquitin chains [7–10]. USPs regulate ubiquitination-meditated degradation and stabilize proteins to maintain the response to signals and altered environmental conditions. This stabilization is established when the deubiquitinating enzymes remove ubiquitin from ubiquitin-conjugated protein substrates to regulate the stability of a target protein. For example, ubiquitin-specific proteases 11 (USP11) have been known as a pro-inflammatory mediator and regulator of apoptosis, proliferation, cancer chemoresistance, ferroptosis, and autophagy [11–14]. However, little is known about the role and mechanisms of USP11 in PAH pathogenesis.

Previous studies demonstrated that transcriptome analysis of PAH lungs reveals expression patterns specific to PAH subtypes, clinical parameters, and lung pathology variables [15,16]. Built on these datasets, we identified associations between survival and single nucleotide polymorphisms (SNPs) in or near genes thought to be relevant to PAH pathogenesis. In particular, we discovered that histidine triad nucleotide binding protein 3 (HINT3, a nucleotide hydrolase and transferase) shows a strong association with worsened survival in PAH. However, the expression, function, and disease-specific action of HINT3 are still unknown. Further, we found that USP11 and HINT3 are strongly associated with PAH. However, the mechanisms and function of the USP11/HINT3 axis are poorly understood in PAH. Therefore, we hypothesized that USP11 activation stabilizes HINT3 to mediate endothelial anti-apoptosis in PAH.

During PAH pathogenesis, pulmonary artery endothelial cells become hyperproliferative and resistant to apoptosis, a programed cell death mechanism that prevents uncontrolled cell replication. Resistance to apoptosis leads to neointimal thickening and narrowing of the vessel lumen [17–19]. B-cell lymphoma 2 (BCL2) in the mitochondria membranes prevents the release of apoptogenic factors and promotes cell survival by blocking apoptosis [20–22]. We also observed increases in BCL2 levels in endothelial cells harvested from the tips of discarded Swan-Ganz catheters after right catheterization in patients with PAH [23], supporting the contribution of BCL2 to the regulation of anti-apoptosis in PAH. From this, we hypothesized that USP11/HINT3 axis regulates the activity and the abundance of the BCL2 proteins, which could lead to cell proliferation of pulmonary arteries in PAH.

In this study, we explored the role and interaction of USP11 and HINT3 in PAH. We show that the levels of USP11 and HINT3 are increased in the lungs of IPAH patients and hypoxia/Sugen-treated mice. Further, we demonstrate that USP11 enhances the stability of HINT3 by deubiquitinating HINT3 and thereby increases anti-apoptotic marker BCL2 levels. Our findings suggest that inhibition of USP11/HINT3 axis may act as a novel therapeutic target in PAH pathogenesis.

## 2. Materials and Methods

### 2.1. Control and IPAH Lung Tissues

We purchased de-identified peripheral lung tissues acquired from patients with idiopathic PAH (IPAH) (2 males and 3 females; 29–55 years old) and healthy individuals (2 males and 2 females; 24–56 years old) collected by the Pulmonary Hypertension Breakthrough Initiative (PHBI, www.phbi.org (accessed on 9 November 2018)).

### 2.2. Reagents

Human pulmonary artery endothelial cells (HPAECs) were obtained from Cell Applications, Inc. (Cell Applications, San Diego, CA, USA). BCL2, GAPDH, and β-actin (ACTB) antibodies were obtained from Cell Signaling Technology (Danvers, MA, USA). USP11 and HINT3 antibodies were purchased from Abcam (Waltham, MA, USA). Fetal bovine serum (FBS) and dimethyl sulfoxide (DMSO) were purchased from Thermo Fisher Scientific (Waltham, MA, USA). The USP11 inhibitor, mitoxantrone, was obtained from Sigma Aldrich (St. Louis, MO, USA).

### 2.3. In Vivo Mouse Model of PH

Ten 8–12 weeks old male C57BL/6J mice were treated with subcutaneous injection of Sugen 5416 (SU, 20 mg/kg) three times a week for 3 weeks. Then, five of them were exposed to hypoxia with an influx of N_2_ gas and the other five to normoxia for 3 weeks, as previously described [24]. All animals had unrestricted access to water and a standard rodent diet. All animal studies were approved by the Institutional Animal Care and Use Committee of Emory University or the Atlanta Veterans Affairs Healthcare System.

### 2.4. In Vitro Human Pulmonary Artery Endothelial Cells

HPAECs were cultured in Endothelial Cell Growth Media (Cell Applications, San Diego, CA, USA) containing 1% (vol/vol) penicillin/streptomycin. Cells were grown under standard incubator conditions at 37 °C and with 5% CO_2_. HPAECs cells were plated in 6-well plate or 10 cm cell culture dishes. Media was changed every 3 days, and cells were split when the cell density reached 80–90% confluence.

### 2.5. Gain or Loss of USP11 or HINT3 Function in HPAECs

For loss of USP11 or HINT3 function (LOF), HPAECs were transfected with scrambled or USP11 (F:5′-GUCAAUGAGAAUCAGAUCGAGUCCA-3′, R:3′-GACAGUUACUCUUAGUCUAGCUCAGGU-5′) or HINT3 (F:5′-CUUGCAGUGAUAUAAUUGACAACAT-3′, R:3′-UUGAACGUCACUAUAUUAACUGUUGUA-5′) dicer-substrate siRNA (DsiRNA) (20 nM, Integrated DNA Technologies, Coralville, IA, USA) using Lipofectamine 3000 transfection reagent (Invitrogen, Carlsbad, CA, USA) according to the manufacturer’s instructions. After transfection for 6 h, the transfection media were replaced with EGM containing 5% FBS and incubated at room temperature for 72 h. For gain USP11 and HINT3, Human HINT3 and USP11 [25] were inserted into pcDNA3.1D/His-V5 TOPO vector. In selected experiments, HPAECs were treated with the USP11 inhibitor, mitoxantrone, for 0–4 h. HPAEC lysates were then harvested and examined for USP11, HINT3, and BCL2 levels using Western blot assays. To overexpress USP11 (gain of function), HPAECs were transfected with USP11 plasmid constructs (1 μg, oxUSP11) or empty vector (Mock) we previously reported.24 After transfection for 6 h, media were replaced with fresh 5% FBS EGM, and HPAEC lysates were then harvested and examined for USP11, HIN3, and BCL2 levels using Western blot assays.

### 2.6. Co-Immunoprecipitation

To ascertain the presence of protein-protein interaction between USP11-HINT3 or HINT3-BCL2, we performed co-immunoprecipitation (co-IP) with HINT3 antibody assay in HPAECs. Briefly, for co-IP, cell lysates (1 mg) were incubated/rotated with primary antibody of HINT3 overnight at 4 °C and then were bound to prewashed 40 μL protein A/G agarose (ThermoFisher Scientific, Waltham, MA, USA) at 4 °C for 2 h. The immunoprecipitated complex was washed three times (5 min each) with cold PBS-T (PBS + 1% Triton). The 2X dye with β-ME was added to the beads and heated at 95 °C for 5 min. The immunoprecipitated complex was analyzed by immunoblotting with an enhanced chemiluminescence detection system (Advansta, San Jose, CA, USA). Then, protein samples were evaluated for equal loading, using β-actin or GAPDH antibody.

### 2.7. HINT3 Half-Life and Ubiquitination

To characterize if the HINT3 protein is degraded by ubiquitination, HPAECs were transfected with 1 μg of HA-ubiquitin plasmids (HA-tagged Ubi) or with control plasmids (empty vector), using Lipofectamine 3000 (ThermoFisher Scientific, Waltham, MA, USA). After twenty-four hours, cell lysates were collected in 1X M-PERTM Mammalian Protein Extraction Reagent (ThermoFisher Scientific, Waltham, MA, USA) and then treated by protease/phosphatase inhibitors examined by immunoblotting. β-actin levels were used as an endogenous control. To examine the half-life of USP11, HPAECs were cultured in complete endothelial cell growth media (EGM) with 1% (vol/vol) penicillin/streptomycin. After twenty-four hours, HPAECs were exposed to 20 μg/mL cycloheximide (CHX) in the presence or absence of 20 μM MG132 for 0, 2, 4, or 8 h.

### 2.8. mRNA Quantitative Real-Time Polymerase Chain Reaction (qRT-PCR) Analysis

To measure USP11, HINT3, and BCL2 levels in the lungs of IPAH patients or mouse lungs, total RNAs were isolated using the total RNA isolation kit (Qiagen, Germantown, MD, USA). Human: USP11, forward: 5′-TCCTCAGCCCAGAGTGTTCT-3′, reverse: 5′-CACACACACAGCAGAAGGTACA-3′, HINT3, forward: 5′-AGTGCGTTAAGTTCCCTGATG-3′, reverse: 5′-CCACCCACTCTCAGAAGTGTT-3′, BCL2, forward: 5′-GTGACCGTACAGTCGGGATT-3′, reverse: 5′-GCCGTACAGTTCCACAAAGG-3′ and mouse: Usp11, forward: 5′-CACCCTCTCCTGGTGTCAGT-3′, reverse: 3′-ATCTGAGGTGGGTTTGGTCA-5′ Hint3 forward: 5′-TGACAGCAACTGCGTGTTCT-3′, reverse: 3′-CCGCTGGTTTGATATCTTTG-5′, and BCL2, forward: 5′-GTACCTGAACCGGCATCTG-3′, reverse: 3′-GCTGAGCAGGGTCTTCAGAG-5′ mRNA levels mRNA levels in the same sample were determined and quantified using specific mRNA primers. Human GAPDH, forward: 5′-GCCCAATACGACCAAATCC-3′, reverse: 3′-AGCCACATCGCTCAGACAC-5′ and mouse Gapdh, forward: 5′-AGCTTGTCATCAACGGGAAG-3′, reverse: 3′-TTTGATGTTAGTGGGGTCTCG-5′ mRNA levels were used as an endogenous control.

### 2.9. Western Blot Analysis

All protein homogenates from human and mouse lungs or HPAECs were subjected to Western blot analysis as reported [24]. Primary antibodies were purchased from Abcam (Waltham, MA, USA) and included USP11 anti-rabbit polyclonal antibody (1:250 dilution, Cat #ab109232, 110 kDa), HINT3 Rabbit polyclonal antibody (1:500 dilution, Cat #ab121960, 20 kDa), BCL2 Rabbit polyclonal antibody (1:500 dilution, Cat #ab59348, 26 kDa). GAPDH rabbit polyclonal antibody (1:10,000 dilution, Cat #G9545, 37 kDa) or ACTB Rabbit polyclonal antibody (1:10,000 dilution, Cat #4970, 37 kDa) was purchased from Cell signaling (Danvers, MA, USA). Proteins were visualized using infrared secondary antibodies (1:10,000) using Rad gel doc proprietary software. Relative protein levels were visualized using image Lab software (Bio-Rad, Hercules, CA, USA), quantified Image J software, and normalized to GAPDH or ACTB levels within the same lane.

### 2.10. Statistical Analysis

For all measurements, data were presented as mean ± standard error of the mean (SEM). All data were analyzed using analysis of variance (ANOVA). Post hoc analysis used the Student Neuman Keuls test to detect differences between specific groups. To test for normality, we employed the Shapiro-Wilk test. If the data were not normally distributed, we performed a Mann-Whitney *U* test. Statistical significance was defined as *p* < 0.05. Statistical analyses were performed using GraphPad Prism, Version 9.0 software (LaJolla, CA, USA).

## 3. Results

### 3.1. USP11 and HINT3 Are Upregulated in the Lung Tissues of PAH Patients and from Hypoxia/Sugen-Treated Mice In Vivo

**Figure 1.**
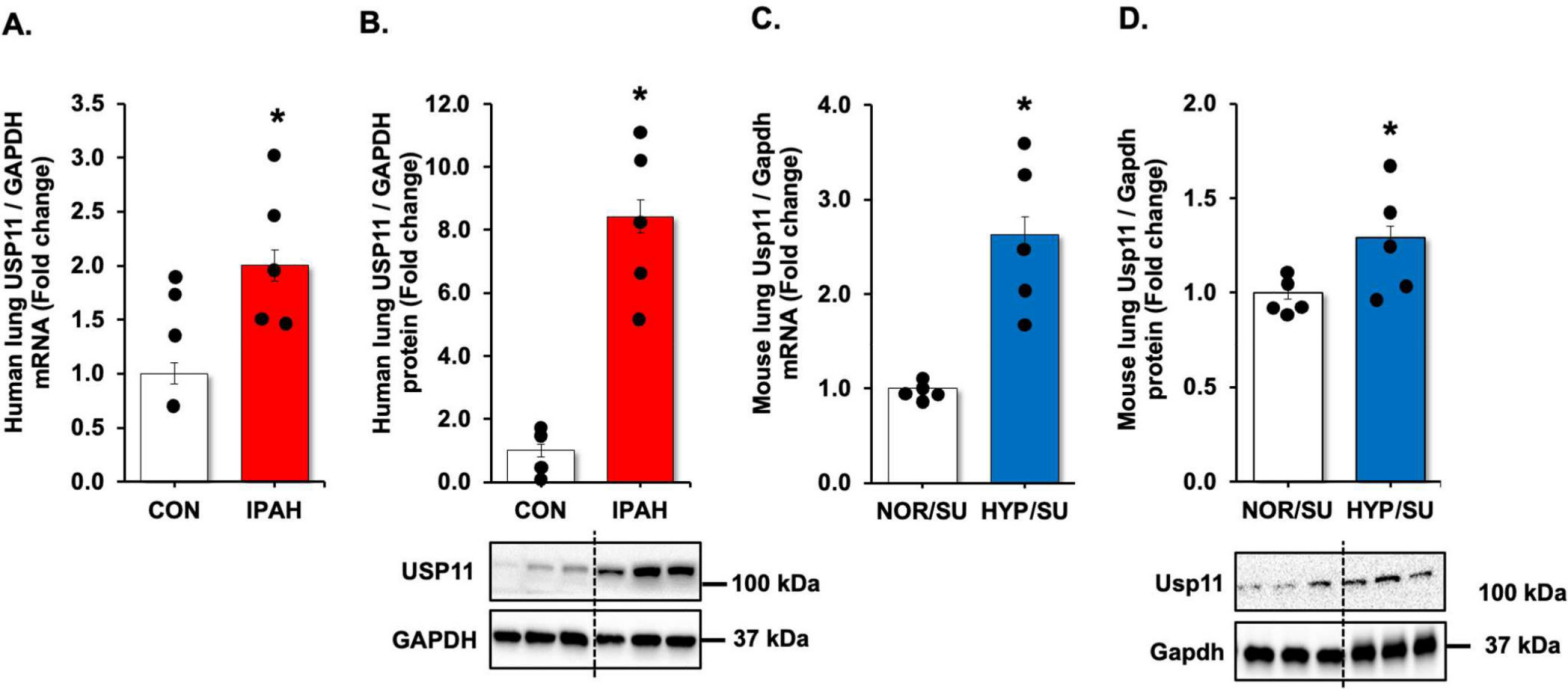
UPS11 is increased in clinical and experimental PAH samples. (**A**,**B**) Fail donor (CON) or idiopathic pulmonary arterial hypertension (IPAH) patient lungs were subjected to qRT-PCR and Western blots. *n* = 4–5. Densitometry with a representative blot and GAPDH loading control is shown in Figure 1B. (**C,D**) Whole lung homogenates were collected from normoxic/sugen (NOR/SU) or hypoxic/Sugen (HYP/SU)-treated mice. Densitometry with a representative blot and GAPDH loading control is shown in Figure 1D. All bars represent mean USP11 mRNA or protein levels ± SEM relative to GAPDH expressed as fold-change *vs.* control (CON) or *vs.* NOR/SU. *n* = 5. * *p* < 0.05 *vs.* respective control condition.

**Figure 2.**
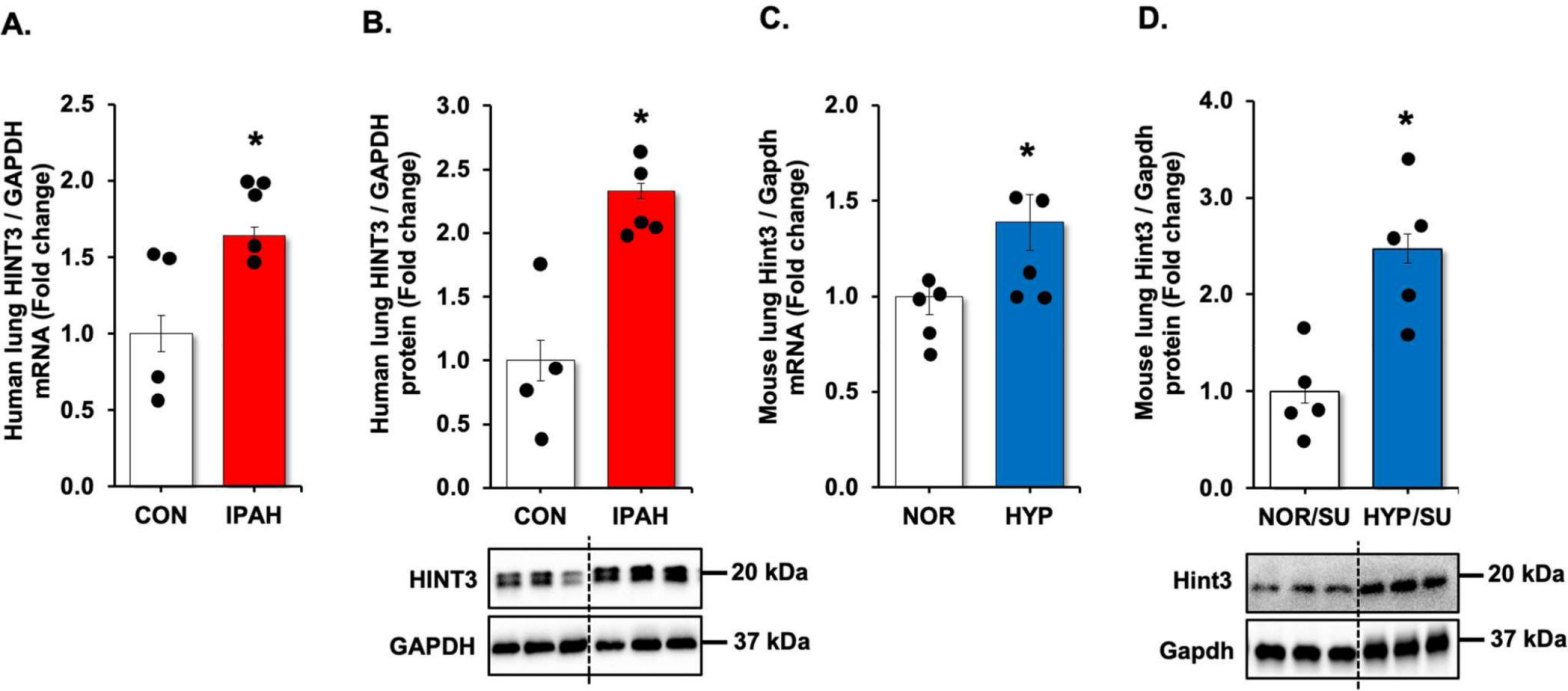
HINT3 is increased in clinical and experimental PAH samples. (**A**,**B**) Fail donor (CON) or idiopathic pulmonary arterial hypertension (IPAH) patient lungs were subjected to qRT-PCR and Western blots. *n* = 4–5. Densitometry with a representative blot and GAPDH loading control is shown in Figure 2B. (**C,D**) Whole lung homogenates were collected from normoxic/sugen (NOR/SU) or hypoxic/Sugen (HYP/SU)-treated mice. Densitometry with a representative blot and GAPDH loading control is shown in Figure 2D. All bars represent mean HINT3 mRNA or protein levels ± SEM relative to GAPDH expressed as fold-change *vs.* control (CON) or *vs.* NOR/SU. *n* = 5. * *p* < 0.05 *vs.* respective control condition.

We revisited our recent genome-wide association study (GWAS) analysis using Affymetrix microarray 15 and found that USP11 and HINT3 were positively correlated, and their expression increased in the lungs of PAH patients compared to controls (Supplementary Figure S1). Therefore, we validated the findings by measuring levels of USP11 and HINT3 mRNAs and proteins in the lung tissues of IPAH patients and hypoxia/Sugen-treated mice. USP11 mRNA (Figure 1A,C) and protein (Figure 1B,D), and concordantly, HINT3 mRNA (Figure 2A,C) and protein (Figure 2B,D), were significantly increased in the lungs of IPAH patients and hypoxia/Sugen-treated mice.

### 3.2. HINT3 Is Degraded by Ubiquitination

**Figure 3.**
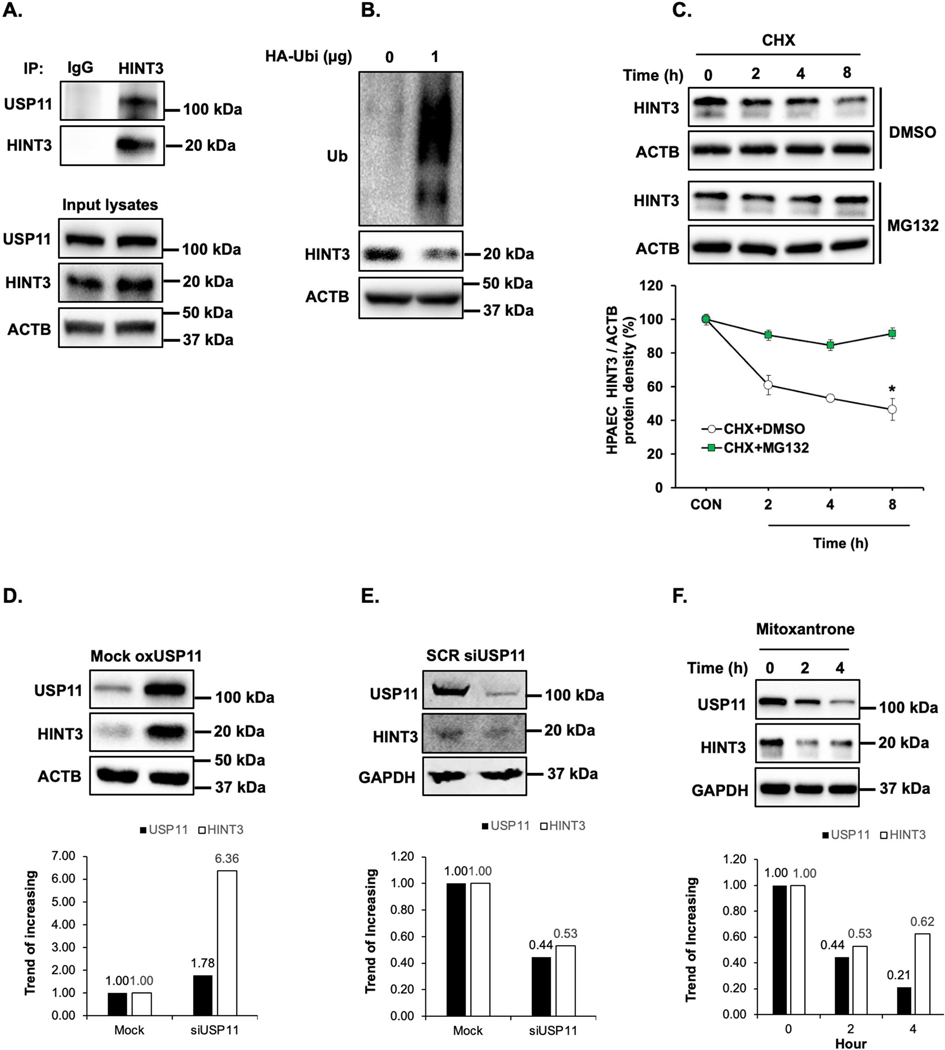
HINT3 is degraded by ubiquitination and USP11 deubiquitinates HINT3 in HPAECs *in vitro*. (**A**) Human pulmonary artery endothelial cells (HPAECs) were cultured for 48 h. Cell lysates were harvested and subjected to immunoprecipitation (IP) with either anti-IgG antibody or anti-HINT3 antibody and then immunoblotted with anti-USP11, anti-HINT3, or anti-β-actin (ACTB) antibody. (**B**) HPAECs were transfected for 24 h with HA-ubiquitin (1 μg, HA-Ubi) plasmid or the control plasmid (empty vector), then collected and assayed by immunoblotting for anti-HA, anti-HINT3, and ACTB (loading control) antibody. (**C**) HPAECs were pretreated with MG132 for 2 h, and then were treated with cycloheximide (CHX, 20 μg/mL) for 0, 2, 4, and 8 h, to inhibit protein synthesis, and harvested for immunoblotting. The level of HINT3 was set as 100% at time 0, and the percent HINT3 protein remaining after CHX treatment at each time point was calculated accordingly. All bars represent mean HINT3 protein levels ± SEM relative to ACTB expressed as fold-change *vs.* CON * *p* < 0.05 *vs.* CON, *n* = 3. (**D**) HPAECs were transfected with USP11 (1 μg, oxHUWE1) plasmid or the control plasmid (empty vector) for 6 h. After media replacement, HPAECs were incubated for additional 72 h. Cell lysates were collected and immunoblotted with anti-HINT3 or ACTB antibody. (**E**) HPAECs were treated with scrambled (SCR) or HINT3 (20 nM) siRNAs for 6 h, media were replaced with endothelial growth medium (EGM) containing 5% FBS. And then incubated for an additional 72 h. Western blotting was performed for HINT3 or GAPDH protein. (**F**) HPAECs were treated with dimethyl sulfoxide (DMSO) or USP11 inhibitor (mitoxantrone) for 0–4 h. Western blotting was performed for HINT3 or GAPDH protein.

To understand the functional significance of the association of USP11 and HINT3, we sought to find protein-protein interactions between USP11 and HINT3 with co-immunoprecipitation (co-IP) assay in HPAECs. As illustrated in Figure 3A, HINT3 strongly binds USP11. Since USP11 has a deubiquitination ability, we hypothesized that HINT3 is degraded by ubiquitination. To address the hypothesis, first, HPAECs were transfected with either control (empty vector) or 1 μg of hemagglutinin (HA)-tagged ubiquitin plasmid (HA-Ubi), and the level of HINT3 was measured. As shown in Figure 3B, HINT3 protein was reduced upon transfection with HA-Ubi, indicating ubiquitin-mediated degradation of HINT3. To further dissect the mechanisms of HINT3 protein degradation, HPAECs were treated in a time-dependent manner with the translational inhibitor, cycloheximide (CHX), to arrest de novo protein synthesis. As illustrated in Figure 3C, CHX treatment revealed a half-life of HINT3 of 8 h. To determine whether HINT3 degradation was primarily dependent on the ubiquitin proteasome pathway, HPAECs were pretreated with the proteasome inhibitor, MG132, followed by CHX. MG132 significantly prolonged the half-life of HINT3 protein, with only 10% degradation at 8 h. These data suggest ubiquitination is a major regulatory step governing steady-state HINT3 levels.

### 3.3. USP11 Deubiquitinates HINT3 in HPAECs In Vitro

Having established that USP11 interacts with HINT3, and that HINT3 degradation is regulated by ubiquitination, we tested whether USP11 acts as a deubiquitinase to stabilize HINT3. First, HPAECs were transfected with USP11 plasmid or empty vector (mock) for 72 h. Overexpression of USP11 increased HINT3 levels (Figure 3D). In parallel, the knockdown of USP11 with siRNA reduced HINT3 expression in HPAECs (Figure 3E). On the other hand, mitoxantrone is a known topoisomerase II inhibitor and is clinically used to decrease cell viability through apoptosis pathway in cancer cells. Thus, the inhibition of USP11 is not the only role of mitoxantrone, and it may have off-target effects [26]. We further explored whether pharmacologic inhibition of USP11 using mitoxantrone affected HINT3 expression. As shown in Figure 3F, mitoxantrone significantly reduced USP11 and HINT3 expression. Taken together, these findings suggest that USP11 interacts with HINT3 to stabilize through deubiquitination.

### 3.4. USP11 Regulates the Anti-Apoptotic Mediator BCL2 via HINT3 Binding Activity

**Figure 4.**
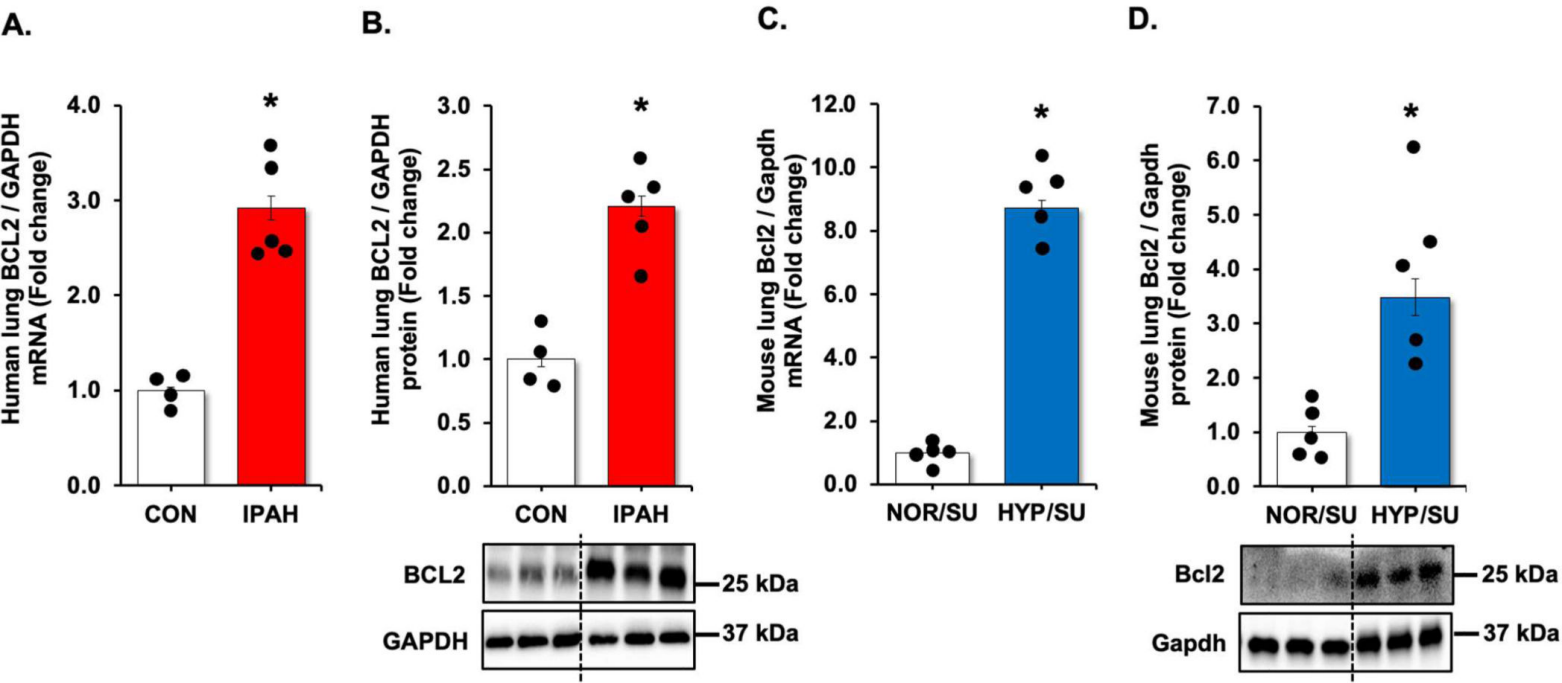
BCL2 is increased in clinical and experimental PH samples. (**A**,**B**) Fail donor (CON) or idiopathic pulmonary arterial hypertension (IPAH) patient lungs were subjected to qRT-PCR and Western blots. *n* = 4–5. Densitometry with a representative blot and GAPDH loading control is shown in Figure 4B. (**C,D**) Whole lung homogenates were collected from normoxic/sugen (NOR/SU) or hypoxic/sugen (HYP/SU)-treated mice. Densitometry with a representative blot and GAPDH loading control is shown in Figure 4D. All bars represent mean BCL2 mRNA or protein levels ± SEM relative to GAPDH expressed as fold-change *vs.* control (CON) or *vs.* NOR/SU. *n* = 5. * *p* < 0.05 *vs.* respective control condition.

**Figure 5.**
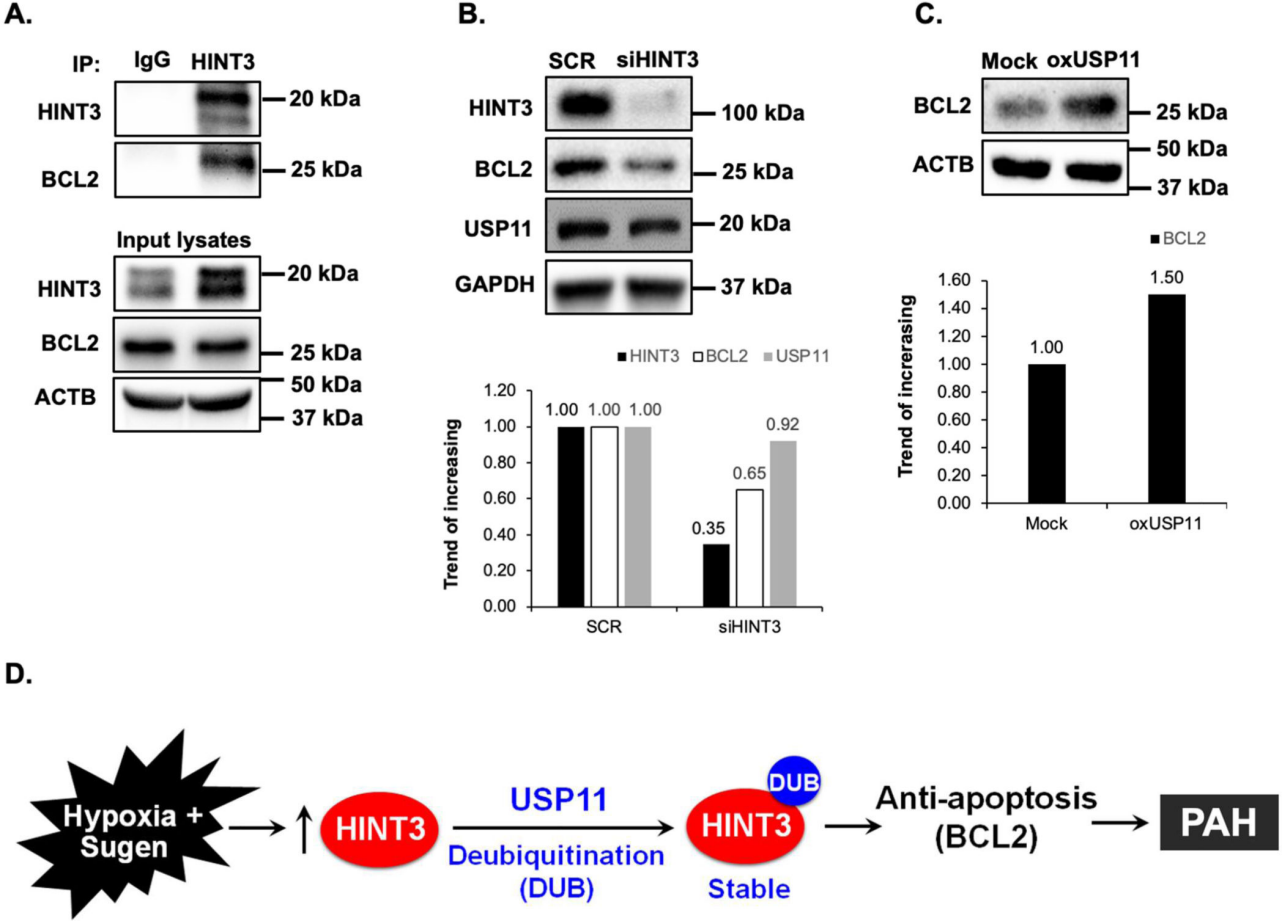
HINT3 regulates BCL2 expression in HPAECs *in vitro*. (**A**) Human pulmonary artery endothelial cells (HPAECs) were cultured for 48 h. Cell lysates were harvested and subjected to immunoprecipitation (IP) with either anti-IgG antibody or anti-HINT3 antibody, and then immunoblotted with anti-HINT3, anti-BCL2, or anti-β-actin (ACTB) antibody. (**B**) HPAECs were treated with scrambled (SCR) or HINT3 (20 nM) siRNAs for 6 h, and the media was replaced with an endothelial growth medium (EGM) containing 5% FBS. And then incubated for an additional 72 h. Western blotting was performed for HINT3, BCL2, USP11, or GAPDH protein. (**C**) HPAECs were transfected with either the control plasmid (Mock) or USP11 (1 μg, oxUSP11) plasmid for 6 h, media were replaced with endothelial growth medium (EGM) containing 5% FBS. And then incubated for an additional 72 h. Cell lysates were collected and immunoblotted with anti-BCL2 or ACTB antibody. (**D**) Hypothetical schema defining the role of USP11/HINT3/BCL2 signaling in PAH pathogenesis. Hypoxia induces USP11, which stabilizes HINT3 levels through HINT3 deubiquitination. Increases in HINT3 stimulate anti-apoptosis marker, BCL2 expression promoting PAH pathogenesis

To further clarify the relationship between USP11, HINT3, and BCL2, we first examined BCL2 expression using qRT-PCR and Western blot analysis. Congruent with previous results [27,28], lung tissue levels of BCL2 were increased in IPAH patients (Figure 4A,B) and hypoxia/Sugen-treated mice (Figure 4C,D). Next, we performed co-IP to determine whether BCL2 interacts with HINT3 and found the interaction between BCL2 and HINT3 (Figure 5A). Interestingly, siRNA knockdown of HINT3 attenuated BCL2 expression in HPAECs, while USP11 levels remained unchanged (Figure 5B). Conversely, overexpression of USP11, which can indirectly increase the expression of HINT3, significantly augmented the expression of BCL2 (Figure 5C). These findings suggest that USP11 deubiquitinates HINT3, allowing it to regulate BCL2 expression positively.

## 4. Discussion

In this work, we discovered regulatory relationships between USP11 and HINT3 associated with apoptosis-resistance in PAH. This shows a mechanistic interplay between novel candidate genes identified in our recent genome-wide association study of PAH. Our findings can be summarized as follows: (1) both USP11 and HINT3 are regulated at multiple levels, based on concordant elevation of mRNA and protein in the lungs of IPAH patients and hypoxia/Sugen-treated mice with experimental PAH *in vivo*; (2) however, the balance of ubiquitination and USP11-mediated deubiquitination is a major determinant of HINT3 protein stability; and (3) HINT3 positively regulates the anti-apoptotic mediator BCL2, consistent with the stereotyped finding of apoptosis-resistance in pulmonary artery endothelial cells. Immunoprecipitation assays corroborate interactions between relevant binding partners: USP11-HINT3-BCL2. Therefore, abnormal upregulation of USP11 contributes to hypo-ubiquitination and inappropriate stabilization of HINT3, resulting in downstream activation of BCL2.

The homeostasis of many cellular proteins is regulated by ubiquitin-mediated proteostasis in response to environmental stimuli. Ubiquitination is a prolific post-translational modification that regulates diverse processes by branding proteins for degradation either by the proteasome or lysosome [29]. Various ubiquitin ligases have been described in neoplastic and degenerative disease processes [30]. Conversely, the removal of ubiquitin chains from ubiquitinated proteins is catalyzed by deubiquitinating enzymes (DUBs), which can rescue substrate proteins from degradation [29]. Abnormal ubiquitination of several vasoactive, redox, and mitogenic proteins such as calveolin-1, angiotensin converting enzyme-2, and superoxide dismutase-2, followed by increased proteasomal degradation, have been reported in PAH [31–33]. Recent studies revealed that inhibition of deubiquitinase (USP15 and UCHL1) reduced cell proliferation and migration of endothelial and smooth muscle cells via YAP/TAZ and AKT1 signaling, respectively, and attenuated PH and PAH [34,35].

Recently, our group demonstrated a global change in the hypoxic lung ‘ubiquitome’, with differential ubiquitination detected in at least 131 proteins [36]. Across the lysine landscape, multiple sites of the proteins were hypo-ubiquitinated, which may also indicate increased DUB activity stimulated by hypoxia. Therefore, the contribution of DUBs to PAH pathogenesis warrants more careful scrutiny. USP11 is a newly described DUB whose repertoire of targets is unknown. Based on the strength of the association between the pair in our GAWAS dataset, we hypothesized that HINT3 could be a downstream target of USP11.

HINT3 is a poorly characterized protein with an unknown function other than AMP hydrolase activity of uncertain significance. There is a paucity of literature on the relevance of HINT3 to disease, but existing reports indicate high expression in breast cancer, where it strongly correlates with mortality [37], and in hepatocellular carcinoma, where it is associated with apoptosis-resistance in the face of serum starvation [38]. These findings indicate that HINT3 may potentially function as a proto-oncogene. Pursuant to this notion, we demonstrated that HINT3 directly interacts with and positively regulates the anti-apoptotic protein BCL2. These findings represent additional novel insights into the clinical significance of HINT3 in IPAH, a condition which features cancer-like expansion of pulmonary vascular endothelial cells. It is worth noting that the effect of the USP11/HINT3 axis may not be limited to pulmonary vascular endothelial cells. Considering that the BCL2 pathway is known to increase viability of smooth muscle cells and fibroblast cells, the effect of the USP11/HINT3 axis on the BCL2 pathway may imply potential effects of USP11/HINT3 on smooth muscle cells and fibroblast in addition to the endothelial cells.

The current study has several important limitations. To confirm the contribution of USP11-HINT3 dysfunction to IPAH, pulmonary artery hemodynamics, right ventricular hypertrophy, and lung vascular remodeling must be directly measured in the endothelial-targeted USP11 overexpressing transgenic mouse model. To solidify the impact of HINT3 on pro-remodeling phenotypes, additional markers of apoptosis, endothelial dysfunction. Proliferation, contractile-secretory transdifferentiation, and migration should be assessed. Cell-type specificity of this pathway, encompassing pulmonary artery smooth muscle cells and adventitial fibroblasts, should similarly be examined. Additionally, to demonstrate the therapeutic feasibility of targeting the USP11-HINT3 axis, studies are needed to investigate whether pharmacologic inhibition or genetic ablation of USP11 attenuates rodent models of PAH. Another limitation of the study is the lack of quantitative analysis of western blot bands, which leaves the western blot analysis as a qualitative representation.

## 5. Conclusions

In summary, the current study mechanically validates the relationship between two novel co-regulated targets detected on genomic and transcriptomic screening. We demonstrate that HINT3 is stabilized through UPS11-directed deubiquitination. Stabilized HINT3 upregulation enhanced BCL2 activation in HPAECs. Our findings prove that mitoxantrone, an FDA-approved antineoplastic agent with canonical DNA topoisomerase inhibitor activity, suppresses USP11 and HINT3 expression. These *in vitro* results lay the groundwork for future studies investigating the drug’s role in vascular biology and its impact on pulmonary hypertension outcomes in *in vivo* mouse models. It may be repurposed or functionalized to target USP11 to restore apoptosis-sensitivity and reduce vascular remodeling in PAH [39]. Furthermore, our results suggest that HINT3 polymorphisms may be prospectively validated in longitudinal patient cohorts as a mechanistic biomarker in PAH.

## Supplementary Material

Supplementary Information

## Data Availability

The statement is required for all original articles which informs readers about the accessibility of research data linked to a paper and outlines the terms under which the data can be obtained.

## References

[1] Galie N, Manes A, Negro L, Palazzini M, Bacchi-Reggiani ML, Branzi A. A meta-analysis of randomized controlled trials in pulmonary arterial hypertension. Eur. Heart J 2009, 30, 394–403. 19155250 10.1093/eurheartj/ehp022 PMC2642921

[2] Lilienfeld DE, Rubin LJ. Mortality from primary pulmonary hypertension in the United States, 1979–1996. Chest 2000, 117, 796–800. 10713009 10.1378/chest.117.3.796

[3] Simonneau G, Montani D, Celermajer DS, Denton CP, Gatzoulis MA, Krowka M, Haemodynamic definitions and updated clinical classification of pulmonary hypertension. Eur. Respir. J 2019, 24, 53. 10.1183/13993003.01913-2018 PMC6351336 30545968

[4] Benza RL, Miller DP, Barst RJ, Badesch DB, Frost AE, McGoon MD. An evaluation of long-term survival from time of diagnosis in pulmonary arterial hypertension from the REVEAL Registry. Chest 2012, 142, 448–456. 22281797 10.1378/chest.11-1460

[5] Badesch DB, Raskob GE, Elliott CG, Krichman AM, Farber HW, Frost AE, Pulmonary arterial hypertension: baseline characteristics from the REVEAL Registry. Chest 2010, 137, 376–387. 19837821 10.1378/chest.09-1140

[6] McGoon MD, Krichman A, Farber HW, Barst RJ, Raskob GE, Liou TG, Design of the REVEAL registry for US patients with pulmonary arterial hypertension. Mayo. Clin. Proc 2008, 83, 923–931. 18674477 10.4065/83.8.923

[7] Chen M, Ding Z, Zhang F, Shen H, Zhu L, Yang H, A20 attenuates hypoxia-induced pulmonary arterial hypertension by inhibiting NF-kappaB activation and pulmonary artery smooth muscle cell proliferation. Exp. Cell. Res 2020, 390, 111982. 32234376 10.1016/j.yexcr.2020.111982

[8] Hao X, Ma C, Chen S, Dang J, Cheng X, Zhu D. Reverse the down regulation of miR-92b-3p by hypoxia can suppress the proliferation of pulmonary artery smooth muscle cells by targeting USP28. Biochem. Biophys. Res. Commun 2018, 503, 3064–3077. 30149918 10.1016/j.bbrc.2018.08.095

[9] Koudstaal T, van Hulst JAC, Das T, Neys SFH, Merkus D, Bergen IM, DNGR1-Cre-mediated Deletion of Tnfaip3/A20 in Conventional Dendritic Cells Induces Pulmonary Hypertension in Mice. Am. J. Respir. Cell. Mol. Biol 2020, 63, 665–680. 32755457 10.1165/rcmb.2019-0443OC

[10] Zhang W, Qi Y, Wu B. MicroRNA-146–5p Promotes Pulmonary Artery Endothelial Cell Proliferation under Hypoxic Conditions through Regulating USP3. Dis. Mark 2021, 2021, 3668422. 10.1155/2021/3668422 PMC8670967 34917199

[11] Basic M, Hertel A, Bajdzienko J, Bonn F, Tellechea M, Stolz A, The deubiquitinase USP11 is a versatile and conserved regulator of autophagy. J. Biol. Chem 2021, 297, 101263. 34600886 10.1016/j.jbc.2021.101263 PMC8546420

[12] Meng C, Zhan J, Chen D, Shao G, Zhang H, Gu W, The deubiquitinase USP11 regulates cell proliferation and ferroptotic cell death via stabilization of NRF2 USP11 deubiquitinates and stabilizes NRF2. Oncogene 2021, 40, 1706–1720. 33531626 10.1038/s41388-021-01660-5

[13] Zhang X, Liu T, Xu S, Gao P, Dong W, Liu W, A pro-inflammatory mediator USP11 enhances the stability of p53 and inhibits KLF2 in intracerebral hemorrhage. Mol. Ther. Methods Clin. Dev 2021, 21, 681–692. 34141823 10.1016/j.omtm.2021.01.015 PMC8178085

[14] Zhu X, Zhang Y, Luo Q, Wu X, Huang F, Shu T, The deubiquitinase USP11 promotes ovarian cancer chemoresistance by stabilizing BIP. Signal. Transduct. Target. Ther 2021, 6, 264. 34257276 10.1038/s41392-021-00580-w PMC8277857

[15] Stearman RS, Bui QM, Speyer G, Handen A, Cornelius AR, Graham BB, Systems Analysis of the Human Pulmonary Arterial Hypertension Lung Transcriptome. Am. J. Respir. Cell. Mol. Biol 2019, 60, 637–649. 30562042 10.1165/rcmb.2018-0368OC PMC6543748

[16] Benza RL, Gomberg-Maitland M, Demarco T, Frost AE, Torbicki A, Langleben D, Endothelin-1 Pathway Polymorphisms and Outcomes in Pulmonary Arterial Hypertension. Am. J. Respir. Crit. Care Med 2015, 192, 1345–1354. 26252367 10.1164/rccm.201501-0196OC PMC4731699

[17] Bhagwani AR, Farkas D, Harmon B, Authelet KJ, Cool CD, Kolb M, Clonally selected primitive endothelial cells promote occlusive pulmonary arteriopathy and severe pulmonary hypertension in rats exposed to chronic hypoxia. Sci. Rep 2020, 10, 1136. 31980720 10.1038/s41598-020-58083-7 PMC6981224

[18] Macias D, Moore S, Crosby A, Southwood M, Du X, Tan H, Targeting HIF2alpha-ARNT hetero-dimerisation as a novel therapeutic strategy for pulmonary arterial hypertension. Eur. Respir. J 2021, 4, 57. 10.1183/13993003.02061-2019 PMC7930471 32972983

[19] Van der Feen DE, Kurakula K, Tremblay E, Boucherat O, Bossers GPL, Szulcek R, Multicenter Preclinical Validation of BET Inhibition for the Treatment of Pulmonary Arterial Hypertension. Am. J. Respir. Crit. Care Med 2019, 200, 910–920. 31042405 10.1164/rccm.201812-2275OC

[20] Cory S, Adams JM. The Bcl2 family: regulators of the cellular life-or-death switch. Nat. Rev. Cancer 2002, 2, 647–656. 12209154 10.1038/nrc883

[21] Tsujimoto Y. Role of Bcl-2 family proteins in apoptosis: apoptosomes or mitochondria? Genes Cells. 1998, 3, 697–707. 9990505 10.1046/j.1365-2443.1998.00223.x

[22] Antico Arciuch VG, Elguero ME, Poderoso JJ, Carreras MC. Mitochondrial regulation of cell cycle and proliferation. Antioxid. Redox. Signal 2012, 16, 1150–1180. 21967640 10.1089/ars.2011.4085 PMC3315176

[23] Benza RL, Williams G, Wu C, Shields KJ, Raina A, Murali S, In situ expression of Bcl-2 in pulmonary artery endothelial cells associates with pulmonary arterial hypertension relative to heart failure with preserved ejection fraction. Pulm. Circ 2016, 6, 551–556. 28090298 10.1086/688774 PMC5210070

[24] Kang BY, Park KK, Kleinhenz JM, Murphy TC, Green DE, Bijli KM, Peroxisome Proliferator-Activated Receptor gamma and microRNA 98 in Hypoxia-Induced Endothelin-1 Signaling. Am. J. Respir. Cell. Mol. Biol 2016, 54, 136–146. 26098770 10.1165/rcmb.2014-0337OC PMC4742924

[25] Wang D, Zhao J, Li S, Wei J, Nan L, Mallampalli RK, Phosphorylated E2F1 is stabilized by nuclear USP11 to drive Peg10 gene expression and activate lung epithelial cells.. Mol. Cell. Biol 2018, 10, 60–73. 10.1093/jmcb/mjx034 PMC6075510 28992046

[26] Bellosillo B, Colomer D, Pons G, Gil J. Mitoxantrone, a topoisomerase II inhibitor, induces apoptosis of B-chronic lymphocytic leukaemia cells. Br. J. Haematol 1998, 100, 142–146. doi:10.1046/j.1365-2141.1998.00520.x 9450803

[27] Cao Y, Jiang Z, Zeng Z, Liu Y, Gu Y, Ji Y, Bcl-2 silencing attenuates hypoxia-induced apoptosis resistance in pulmonary microvascular endothelial cells. Apoptosis 2016, 21, 69–84. 26456506 10.1007/s10495-015-1184-3

[28] Hu CJ, Zhang H, Laux A, Pullamsetti SS, Stenmark KR. Mechanisms contributing to persistently activated cell phenotypes in pulmonary hypertension. J. Physiol 2019, 597, 1103–1119. 29920674 10.1113/JP275857 PMC6375873

[29] Wolberger C. Mechanisms for regulating deubiquitinating enzymes. Prot. Sci 2014, 23, 344–353. 10.1002/pro.2415 PMC3970886 24403057

[30] Hochstrasser M. Biochemistry. All in the ubiquitin family. Science 2000, 289, 563–564. 10939967 10.1126/science.289.5479.563

[31] Bakhshi FR, Mao M, Shajahan AN, Piegeler T, Chen Z, Chernaya O, Nitrosation-dependent caveolin 1 phosphorylation, ubiquitination, and degradation and its association with idiopathic pulmonary arterial hypertension. Pulm. Circ 2013, 3, 816–830. 25006397 10.1086/674753 PMC4070841

[32] Schultz A, Olorundami OA, Teng RJ, Jarzembowski J, Shi ZZ, Kumar SN, Decreased OLA1 (Obg-Like ATPase-1) Expression Drives Ubiquitin-Proteasome Pathways to Downregulate Mitochondrial SOD2 (Superoxide Dismutase) in Persistent Pulmonary Hypertension of the Newborn. Hypertension 2019, 74, 957–966. 31476900 10.1161/HYPERTENSIONAHA.119.13430 PMC6739165

[33] Shen H, Zhang J, Wang C, Jain PP, Xiong M, Shi X, MDM2-Mediated Ubiquitination of Angiotensin-Converting Enzyme 2 Contributes to the Development of Pulmonary Arterial Hypertension. Circulation 2020, 142, 1190–1204. 32755395 10.1161/CIRCULATIONAHA.120.048191 PMC7497891

[34] Wu Z, Zhu L, Nie X, Wei L, Qi Y. USP15 promotes pulmonary vascular remodeling in pulmonary hypertension in a YAP1/TAZ-dependent manner. Exp. Mol. Med 2023, 55, 183–195. doi:10.1038/s12276-022-00920-y 36635430 PMC9898287

[35] Tang H, Gupta A, Morrisroe SA, Bao C, Schwantes-An TH, Gupta G, Deficiency of the Deubiquitinase UCHL1 Attenuates Pulmonary Arterial Hypertension. Circulation 2024, 150, 302–316. doi:10.1161/CIRCULATIONAHA.123.065304. 38695173 PMC11262989

[36] Wade BE, Zhao J, Ma J, Hart CM, Sutliff RL. Hypoxia-induced alterations in the lung ubiquitin proteasome system during pulmonary hypertension pathogenesis. Pulm. Circ 2018, 8, 2045894018788267. 29927354 10.1177/2045894018788267 PMC6146334

[37] Uhlen M, Fagerberg L, Hallstrom BM, Lindskog C, Oksvold P, Mardinoglu A, Proteomics. Tissue-based map of the human proteome. Science 2015, 347, 1260419. 25613900 10.1126/science.1260419

[38] Wang W, Xu G, Ding CL, Zhao LJ, Zhao P, Ren H, All-trans retinoic acid protects hepatocellular carcinoma cells against serum-starvation-induced cell death by upregulating collagen 8A2. FEBS J. 2013, 280, 1308–1319. 23298258 10.1111/febs.12122

[39] Burkhart RA, Peng Y, Norris ZA, Tholey RM, Talbott VA, Liang Q, Mitoxantrone targets human ubiquitin-specific peptidase 11 (USP11) and is a potent inhibitor of pancreatic cancer cell survival. Mol. Cancer. Res 2013, 11, 901–911. 23696131 10.1158/1541-7786.MCR-12-0699

