# Supplementary material for: USP11 Promotes Endothelial Apoptosis-Resistance in Pulmonary Arterial Hypertension by Deubiquitinating HINT3
Source: J Respir Biol Transl Med. Author manuscript; Available in PMC 2025 May 15. (PMC12080269; doi:10.70322/jrbtm.2025.10002)
Supplement: Supplementary Information [file NIHMS2069919-supplement-Supplementary_Information.pdf]

Supplementary Figure S1

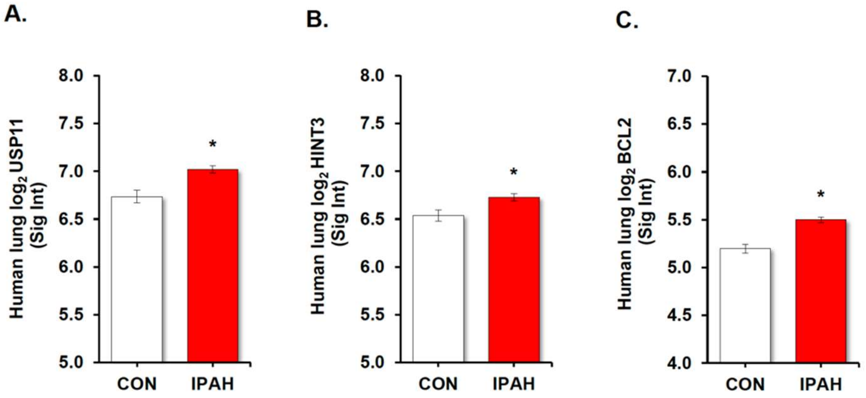

Supplementary Figure S1. PAH exhibits upregulated USP11, HINT3, and BCL2 expression. RNA sequencing was conducted using PAH human vs failed donor normal lung. Data are expressed as mean USP11, HINT3, or BCL2 ± SE.
